# The effects of a sugar-free amino acid-containing electrolyte beverage on 5-kilometer performance, blood electrolytes, and post-exercise cramping versus a conventional carbohydrate-electrolyte sports beverage and water

**DOI:** 10.1080/15502783.2023.2296888

**Published:** 2023-12-22

**Authors:** Mason C. McIntosh, Bradley A. Ruple, Nicholas J. Kontos, Madison L. Mattingly, Christopher M. Lockwood, Michael D. Roberts

**Affiliations:** a Nutrabolt Applied and Molecular Sciences Laboratory, Auburn University, School of Kinesiology, Auburn, AL, USA; b Nutrabolt, Austin, TX, USA; c Dr Chris Lockwood LLC, Casper, WY, USA

**Keywords:** Branched chain amino acids, endurance exercise performance, fatigue, supplementation, time trial

## Abstract

**Objective:**

The purpose of this study was to examine the acute effects of a multi-ingredient, low calorie dietary supplement (MIDS, XTEND® Healthy Hydration) on 5-kilometer (5-km) time trial performance and blood electrolyte concentrations compared to a carbohydrate-electrolyte beverage (CE, GATORADE® Thirst Quencher) and distilled water (W).

**Methods:**

During visit 1 (V1), participants (10 men and 10 women, 20–35 years old, BMI ≤ 29 kg/m^2^, recreationally active) reported to the laboratory whereby the following tests were performed: i) height and weight measurements, ii) body composition analysis, iii) treadmill testing to measure maximal aerobic capacity, and iv) 5-km time trial familiarization. The second visit (V2) was one week after V1 in the morning (0600 – 0900) and participants arrived 12–14 h fasted (no food or drink). The first battery of assessments (V2-T1) included nude body mass, urine specific gravity (USG), a profile of mood states (POMS) questionnaire, and the completion of a visual analogue scale (VAS) questionnaire to quantify cramping. Then heart rate (HR), blood pressure (BP), total body hydration (via bioelectrical impedance spectroscopy [BIS]) were examined. Finally, a measurement of blood markers via finger stick was performed. Participants consumed a randomized beverage (16 fl. oz. of MIDS, 16 fl. oz. of W, or 16 fl. oz. of CE) within 3 min followed by a 45-min rest. Following the rest period, a second battery (V2-T2) was performed whereby participants’ USG was assessed and they completed the POMS and VAS questionnaires, and HR, BP, and blood markers were measured. The participants then performed a 5-km treadmill time trial. Immediately following the 5-km time trial, participants completed a third testing battery (V2-T3) that began with blood markers, HR and BP assessments, followed by nude body weight assessment, and the POMS and VAS questionnaires. After 60 min, a fourth battery (V2-T4) was performed that included HR, BP, and blood markers. After sitting quietly for another 60 min a fifth battery assessment was performed (V2-T5) that included participants’ USG, POMS and VAS questionnaires, HR, BP, blood markers, and total body hydration. Visits 3 (V3) and 4 (V4) followed the same protocol except a different randomized drink (16 oz. of CE, MIDS, or W) was consumed; all of which were separated by approximately one week.

**Results:**

No differences occurred between conditions for 5-km time trial completion, indirect calorimetry outcomes during 5-km time trials, USG, or nude mass measurements (*p* > 0.05 for all relevant statistical tests). However, blood potassium and the sodium/potassium ratio displayed significant interactions (*p* < 0.05), and post hoc testing indicated these values were better maintained in the MIDS versus other conditions. Post-exercise cramp prevalence was greater in the CE (*p* < 0.05) and trended higher with W (*p* = 0.083) compared to the MIDS condition. Post-exercise cramp severity was also elevated with the W and CE beverages (*p* < 0.05) but not the MIDS (*p* = 0.211).

**Conclusions:**

The MIDS did not affect 5-km time trial performance but exhibited favorable effects on blood electrolyte and post-exercise self-reporting cramp outcomes compared to the CE and W drinks.

## 1. Background

Many athletes today utilize ergogenic aids to maximize their performance or enhance recovery from exercise, as an estimated 50% of American adults routinely use dietary supplements [1,2]. Commercially available, multi-ingredient dietary supplements (MIDS) have gained popularity for their potential ergogenic effects on exercise performance. Sugar-free, electrolyte-containing hydration MIDS beverages have gained incredible market traction. In this regard, the market for such beverages was valued at $24.4 billion in 2021 and is expected to reach $32.6 billion by 2027 [3].

Dehydration is a limiting factor for aerobic activity performance [4], and consuming carbohydrate-electrolyte beverages have served as an adjuvant for their ability to maintain blood glucose and muscle glycogen stores while replacing electrolytes and fluids that are lost during exercise [5]. To this end, there are several studies that have shown carbohydrate-only or carbohydrate-electrolyte (CE) beverages extend aerobic performance and/or are ergogenic [6–10]. However, endurance exercise performance can be optimized through blends of amino acids given their involvement in cellular metabolism, particularly during exercise [11]. Additionally, amino acids can serve a fluid volume-regulatory function as amino acids provide intracellular support therefore allowing ion concentrations to remain constant during physiologically stressful conditions [12]. Manufacturers who formulate MIDS aim to compete with market standard carbohydrate-electrolyte beverages by producing products with novel ingredient combinations and doses for a proposed synergistic effect that will maximize exercise performance [13]. Further, a current MIDS, XTEND® (Healthy Hydration; Nutrabolt, Austin, TX, USA), may be ergogenic in this regard due to its electrolyte and amino acid profile, and the osmolyte betaine (trimethylglycine).

Using a crossover design in recreationally-active younger adults, we sought to explore the effects of a sugar-free, electrolyte MIDS beverage (XTEND® Healthy Hydration; Nutrabolt, Austin, TX, USA) on 5-kilometer (5-km) time trial performance, hydration markers, blood electrolytes and metabolites, fuel oxidation, and perceptions of mood compared to a standard commercially available carbohydrate sports beverage (“CE,” GATORADE® Thirst Quencher; PepsiCo, Purchase, NY, USA) and distilled water (W) as a positive control. Based on the literature discussed above, we hypothesized that the MIDS and CE (versus W) would improve 5-km time trial performance in addition to better maintaining blood electrolytes. We also hypothesized that MIDS would not outperform CE in affecting these variables. Finally, while our 5-km time trial occurred in ambient laboratory conditions, we sought to explore how post-exercise cramping was affected. Given that this was a secondary outcome, we did not adopt an a priori hypothesis.

## 2. Methods

### 2.1. Participants and ethical approval

This study was conducted with prior review and approval from Auburn University Institutional Review Board and in accordance with the most recent revisions of the Declaration of Helsinki (IRB approval #:22–513 MR 2212).

Young adults were recruited from the local community, meeting the following criteria: i) between the ages of 20–35 years old, ii) Body mass index (BMI) less than 29 kg/m^2^, iii) Actively participating in any structured training program (≥2 days/week) for the past 6 months, iv) Free of any known orthopedic condition, cardiovascular or metabolic disease that would contraindicate running with vigor on a treadmill or donating blood, vi) Not allergic to contents in the MIDS or CE drinks, vii) Does not consume ≥ 21 servings of caffeine (serving: 170.5 mL) per week [14], viii) Does not abuse alcohol, use illicit drugs, or take drugs and/or medications that are intended to affect outcomes related to ADD/ADHD, depression, or sleep. Following verbal and written consent, participants completed a maximal oxygen uptake treadmill test to ensure that participants met the fitness criteria. VO_2_max criteria needed to qualify was 44.9–66.3 ml/kg/min for 18–29-year-old males, 34.6–56.0 ml/kg/min for 18–29-year-old females, 39.6–59.8 ml/kg/min for 30–35-year-old males, and 28.2–45.8 ml/kg/min for 30–35-year-old females. These criteria were based upon the ACSM’s Cardiorespiratory Fitness Classification of not less than “Fair” by age and sex [15].

### 2.2. Experimental design

**Figure 1.**
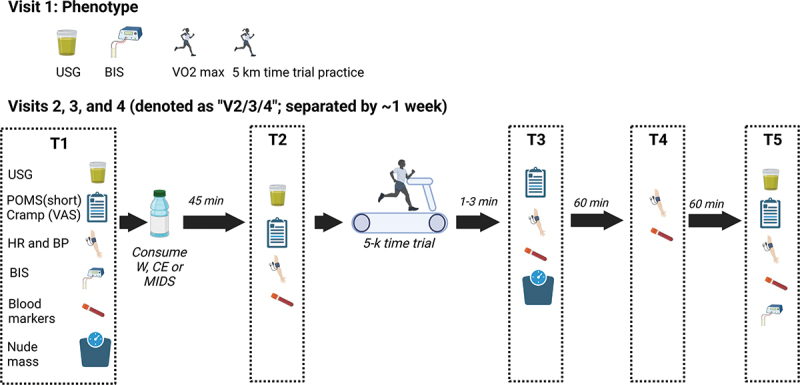
Study design. This schematic (created using Biorender.com) shows the sequence of testing events employed for the current study. Abbreviations: BIS, bioelectrical impedance spectroscopy; BP, blood pressure assessment; HR, heart rate assessment; POMS, profile of mood state questionnaire; USG, urine-specific gravity; VAS, visual analog scale (Wong-baker). Please see Figure 2 for a full breakdown of ingredients included in the MIDS.

**Figure 2.**
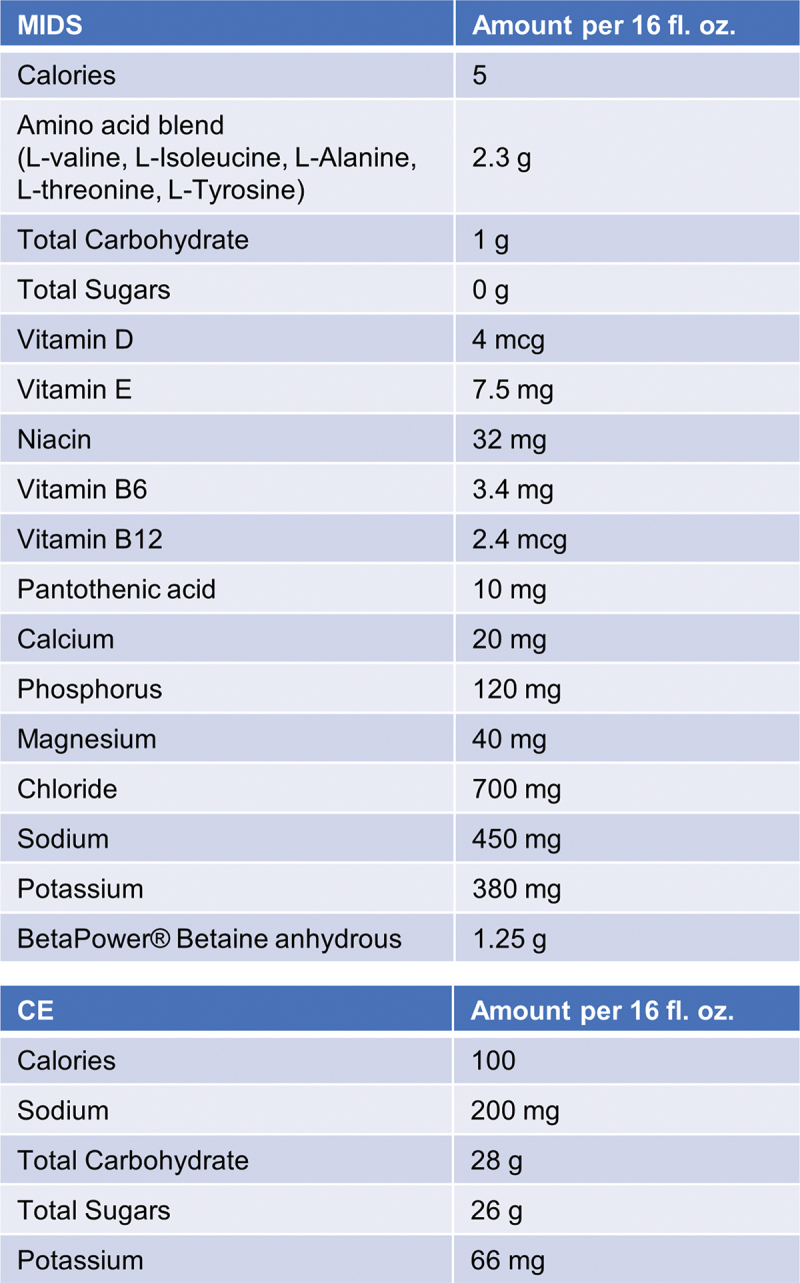
CE and MIDS contents. These contents are based on 16 fl. oz. servings provided to participants during the trials.

The timeline and testing battery for each visit can be visualized in Figure 1 and is described in further detail below. The study was executed whereby supplements were blinded to participants, and visits occurred in a randomized and crossover fashion. Given that the researchers obtained the CE themselves (i.e. the supplement was not provided by the research sponsor), the researchers best attempted to maintain double blindness throughout the trial. These efforts involved: a) one person making the drinks in unlabeled Dixie cups away from the remainder of the research team and participant, and immediately providing it to the participant to drink after preparation occurred, b) the person making the drinks in this fashion (i.e. B.A.R.) not being involved in treadmill testing to minimize influencing performance outcomes and, instead, being involved in drawing finger blood and inserting samples into the blood-gas analysis machine, and c) the data being statistically analyzed by the corresponding author who was not privy to treatments and was unblinded following all statistical analyses. However, it should be noted that participants were aware when consuming W given that it was not flavored. The MIDS and CE were flavor-matched, and third party tested for ingredient integrity. During informal discussions between participants and researchers, it was indicated that the participants were incapable of differentiating between the MIDS and CE condition based on taste. Participants reported to the laboratory on 4 separate occasions with a one-week washout period and each session lasted ~3 hours (see Figure 1). Additionally, please see Figure 2 for a full breakdown of ingredients included in the MIDS.

During visit 1 (V1), participants reported to the laboratory and underwent an array of testing beginning with urine specific gravity (USG) to ensure adequate hydration. The following examinations were completed thereafter: i) height and weight measurements, ii) body composition analysis, iii) treadmill testing using a metabolic testing device (ParvoMedics TrueMax 2400; Sandy, UT, USA) to measure maximal aerobic capacity (VO₂max), and iv) 5-km time trial familiarization while using the metabolic testing device to measure VO₂ during a steady state run pace.

The second visit (V2) took place in the morning (0600 – 0900); participants were asked to arrive after a 12–14 h fast (no food or drink). The first battery of assessments (V2-T1) included nude bodyweight (in a private restroom), USG, an abbreviated profile of mood states (POMS) questionnaire, and the completion of a visual analogue scale (VAS) questionnaire to quantify cramping. Then heart rate (HR), blood pressure (BP), total body hydration (via bioelectrical impedance spectroscopy [BIS]) were examined. Finally, a measurement of blood markers via finger stick was performed using a capillary tube and blood-gas analyzer (GEM Premier 4000 blood gas analyzer; Instrumentation Laboratory, Bedford, MA, USA). The participants were instructed to consume their drink (16 fl. oz. of MIDS, 16 fl. oz. of distilled water, or 16 fl. oz. of CE) within a 3-min period while sitting and resting quietly for 45 mins. Following a 45-min post-treatment rest period, a second battery (V2-T2) was performed whereby participants’ USG was assessed and they completed the abbreviated POMS questionnaire and VAS questionnaires, and HR, BP, and blood markers were measured. The participants then ran a 5-km time trial following a standardized warm-up (3 mins, 3 mph, at 1% grade) while using the metabolic testing device to collect expired gases. HR and participants’ rating of perceived exertion (RPE) was recorded every 3 mins. Immediately following the completion of the 5-km time trial, participants completed a third testing battery (V2-T3) that began with blood markers, HR and BP assessments, followed by nude body weight assessment and the abbreviated POMS and VAS questionnaires. After sitting quietly for 60 mins, a fourth battery (V2-T4) was performed that included HR, BP, and blood markers. After sitting quietly for another 60 mins a fifth battery assessment was performed (V2-T5) that included participants’ USG, POMS and VAS questionnaires, HR, BP, blood markers, and total body hydration (via BIS). The total recovery time following the 5-km time trial until the participant completed the study visit lasted ~130 mins. Visits 3 (V3) and 4 (V4) followed the same timeline with respect to consuming a different randomized drink (16 oz. of CE, MIDS, or W) each visit; all of which were separated by one week. Participants’ visits were scheduled to be at the same time of day (±1 h) for each visit.

### 2.3. Specific testing procedures

*Urine Specific Gravity*. At the beginning of V1 and V2–4, at timepoints T1, T2, and T5, participants donated approximately ~5 mL of urine which was analyzed with a handheld master refractometer (ATAGO®, Japan) for USG levels.

#### 2.3.1. Body composition and total body water assessments

Body composition and whole-body hydration were assessed during V1 and V2–4, at timepoints T1 and T5. Following USG assessments, body mass and height were assessed using a laboratory scale (Seca, Chino, CA, USA). Participants then stood on a SOZO® BIS platform (ImpediMed, Carlsbad, CA, USA) to assess body composition, total body water (TBW), extracellular fluid (ECF), and intracellular fluid (ICF). Prior to the test, participants were asked to remove any metallic objects and footwear (i.e. shoes, socks, etc.). The participants were then asked to grasp the handles on the SOZO® and remain still and quiet while the device was operating.

#### 2.3.2. V1 VO2max assessment and practice run for 5-km pacing

During V1, participants were attached to a metabolic testing device using a mask that covered the nose and mouth and were asked to step on the laboratory treadmill (Woodway; Waukesha, WI, USA). The maximal test used was modified from Gaddie et al. [16]. First, a 3-min walking warm-up was performed at 3.0 mph (1% grade). Next, stage 1 consisted of one of 3 starting speeds chosen based on the participant’s perceived capabilities (5.0, 5.5, 6.0 mph). This speed was performed for 2 min at 1% incline. Thereafter, the speed was increased 1 min/mile for 2-min stages (i.e. Stages 2/3/4) until stage 5. At stage 5, the treadmill incline increased by 2%, while the speed remained constant. Thereafter, the incline increased every 2 min for stages 5/6/7 until volitional fatigue whereby the participant dismounted the treadmill. Within each stage, the participants’ ratings of perceived exertion (RPE) according to the 6–20 Borg scale were assessed. Real-time heart rates were also assessed using chest strap heart rate monitors (Polar; Kempele, Oulu, Finland).

Approximately 5 min following the VO₂max test, participants were instructed to step back onto the treadmill and reattach themselves to the metabolic testing device and run at a pace mimicking what they would perform during a 5-km time trial. Once participants reached a steady-state running speed (which was confirmed through indirect calorimetry via a plateau of VO_2_ levels) the participants were instructed to cease running. This practice run was ~ 1.0–1.5 km in duration for all participants.

Notably this test also allowed us to obtain ventilatory threshold values for participants, and these data were leveraged during the 5-km runs to determine relative running intensities.

#### 2.3.3. Abbreviated profile of mood states questionnaire

During T1, T2, T3, and T5 of lab visits V2, 3, and 4, participants were instructed to complete the POMS questionnaire to assess the participants’ state of mood before and after exercise [17]. The questionnaire consisted of 40 items with a 4-point scale (“0 = not at all” to “4 = extremely”), and results were tabulated to provide total mood disturbance and vigor scores.

#### 2.3.4. Visual analogue scale for cramping

Participants were instructed to complete the VAS questionnaire each time they completed the POMS questionnaire to assess if cramping had occurred and the severity of such cramping. The Wong-Baker pain scale questionnaire was used to assess if a cramp had occurred and the location of the cramp. The pain scale possessed scores of 0–10 (“0 = no hurt” to “10 = hurts worst”) with corresponding faces to assess the severity of the cramp [18].

#### 2.3.5. Heart rate and blood pressure measurements

These measurements were taken each time the participant arrived for a visit, except for V1. A brachial cuff was applied to the participant’s right arm while they lied in a supine position for 5 min (except for T2 and T3 where measures were taken immediately upon researcher and participant interaction) before measures were taken to assess HR and BP using an automated BP monitor (OMRON, Lake Forest, IL, USA).

#### 2.3.6. Measurement of blood markers

These measurements were taken during each testing battery. While the participant was seated, a researcher used a single-use lancet (SensiLance, Orangeburg, NY, USA) to puncture the participant’s fingertip and collect fresh capillary blood within a 170 μL heparinized plastic capillary tube (GEM Safety Draw, Instrumentation Laboratory Company, Bedford, MA, USA). The capillary blood was immediately analyzed with a blood-gas analyzer (GEM Premier 4000) whereby pH, sodium (Na^+^), potassium (K^+^), hematocrit, glucose, and lactate were assessed. Chloride was not measured due to repeated calibration issues with the blood-gas analyzer.

#### 2.3.7. Nude mass assessment

During V2-T4 for the T1/3, participants were asked to step into a private restroom and on to an electronic scale (Michelli Scales, Mobile, AL, USA), while completely free of any clothing, and report their body mass value to the researcher after exiting the restroom.

#### 2.3.8. Supplement protocol

Following T1 for V2-T4, participants were instructed to drink 16 fl. oz. of CE, 16 fl. oz. of W, or 16 fl. oz. of MIDS within 3 min of receiving the drink. The drink was randomized and blinded to the participant and prepared by a researcher in an opaque cup. Bottled W (Ecoxall Chemicals 100% Pure All Natural Deionized Water; Brighton, MI, USA) and CE (GATORADE® Thirst Quencher; PepsiCo, Purchase, NY, USA) were purchased from an online retailer, and MIDS (XTEND® Healthy Hydration; Nutrabolt®, Austin, TX, USA) was modified for organoleptics to match CE for blinding and manufactured at a US FDA cGMP-compliant facility. Random CE and MIDS bottles were sent by the researchers for independent, third-party laboratory (DYAD Labs; Merieux NutriSciences Company, Salt Lake City, UT, USA) verification of nutrition facts, physical properties and organoleptics, and specification identity, composition, potency and purity using validated analytical methods. Likewise, random CE and MIDS bottles were also sent for independent, third-party laboratory (Informed Sport; LGC Sciences, Inc., Lexington, KY, USA) verification of the absence of World Anti-Doping Agency (WADA) banned substances using validated methods. These analyses confirmed that CE and MIDS met all analytical specifications (see Figure 2 for finished product Nutrition Facts for CE and Supplement Facts for MIDS) and were free of banned substances. Analytical results were reviewed by C.M.L. prior to the beverages being approved for use within the present study.

#### 2.3.9. 5-kilometer time trials

Time trials were completed 45 min following drink consumption during V2-T4. Participants were interfaced with the metabolic cart using facemasks and asked to step on the treadmill. The treadmill was set to a 1% grade and participants were given a 3-min warm-up where they walked at 3 mph. Following the 3-min warm up, participants self-selected a speed on the treadmill and ran until they completed their 5-km time trial run. The speed of the treadmill and time recorded was covered so the participants would be blinded to the pace at which they were performing. Participants’ RPEs during the trials were recorded every 3 mins, and real-time HR were obtained using wearable heart rate monitors (Polar; Kempele, Oulu, Finland). Once the 5-km distance was obtained, the time to completion was recorded and participants were asked to dismount the treadmill for immediate post-exercise assessments (T3 during V2-T4) as described above.

### 2.4. Statistical analysis

5-km time trial variables (i.e. time-to-completion, VO_2_peak during 5-km trials, mean VO_2_ during 5-km trials, average HR during 5-km trials, and average RPE during 5-km trials) were assessed using repeated measures ANOVAs, and Tukey’s multiple comparisons post hoc tests were used to determine significant effects between treatments.

Other tests (i.e. blood markers, cramping and POMS questionnaire data, hemodynamic measurements, nude mass, and USG) were analyzed using two-way within-within repeated measures ANOVAs. Tukey’s multiple comparisons post hoc tests were used to determine where significance occurred in the event of significant interactions. GraphPad Prism (v9.5.1; Boston, MA, USA) was used for all statistical analyses and to construct graphs, all data are presented as mean ± standard deviation values, and statistical significance was established as *p* < 0.05.

Notably, most dependent variables within each condition yielded data that was mostly normally distributed when examining each time point within each condition. For instance, according to p-values yielded from Shapiro-Wilks tests, 60% of the blood sodium and potassium data was normally distributed, 73–100% of the lactate, pH, calcium, and all hematocrit data, the time to complete the 5 km time trial, and BIS data were normally distributed. Thus, parametric statistics were performed on these data. However, the post-exercise cramp data was consistently non-normally distributed across conditions. Hence, parametric and non-parametric analyses were performed for these variables and p-values are presented in the results accordingly.

## 3. Results

### 3.1. Participant characteristics

Participant characteristics are presented in Table 1. Twenty participants (*n* = 20; male = 10; female = 10; age = 28 ± 4 yrs) completed all trials. One participant had to voluntarily drop due to a knee injury sustained outside of the study that prevented this person from completing his last 5-km time trial. All other 20 participants that finished the trials reported no adverse effects after consuming the CE, W and MIDS drinks.

**Table 1.** Participant characteristics.

Variable	Mean (standard deviation)
Age (years)	28 (4)
Body mass (kg)	71.3 (13.6)
Body fat % (BIS)	22.2 (6.2)
BMI (kg/m^2^)	24.5 (2.8)
VO_2_peak (ml O_2_/kg/min)	46.5 (7.9)
%VO_2_peak during partial 5-km practice	84.2 (7.2)

Legend: These data are for the *n* = 20 finishers (10 men, 10 women). Abbreviation: BIS, bioelectrical impedance spectroscopy.

### 3.2. 5-kilometer time trial variables

**Figure 3.**
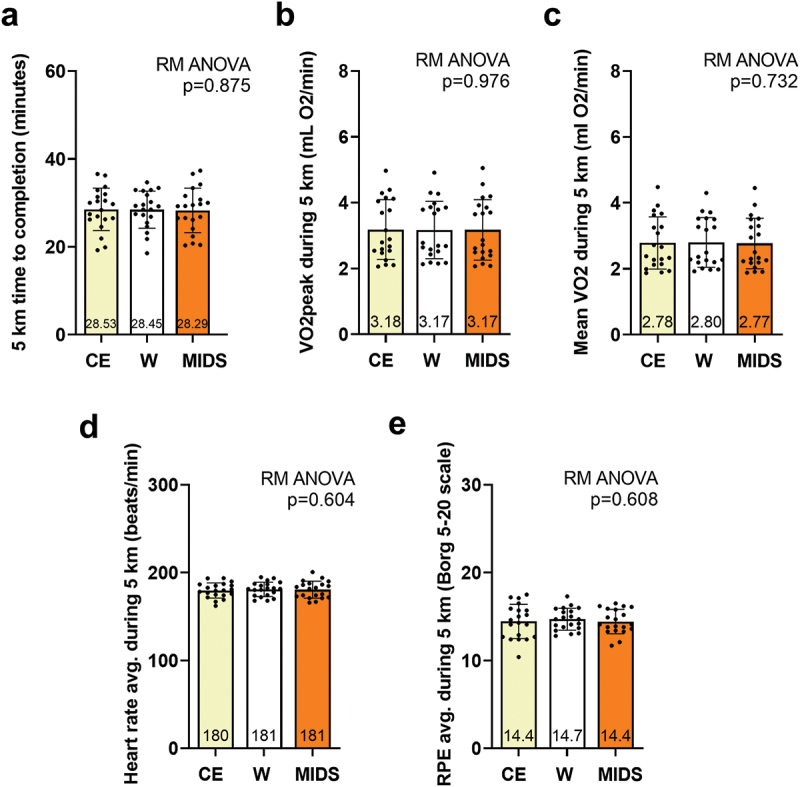
5-km time trial variables. repeated measures (RM) ANOVAs indicated that no significant differences between trials existed for 5 km time to completion (panel a), VO2peak during the time trial (panel b), mean VO2 during the time trial (panel c), average heart rate during the time trial (panel d), or average rating of perceived exertion during the time trial (panel e). All data are presented as mean ± standard deviation values, individual respondent data are presented as data points within each bar, and the mean scores are presented at the bottom of each bar.

Participant performance outcomes are presented in Figure 3. There were no statistical differences between conditions for 5-km time trial performance (Figure 3a), VO_2_peak values during the time trials (Figure 3b), average VO_2_ during the time trials (Figure 3c), average heart rate during the trials (Figure 3d), or average RPE (Figure 3e). Also notable, environmental conditions averaged to be similar between the CE (22.7 ± 1.6ºC, 40.2 ± 12.3 % humidity), W (22.8 ± 1.2ºC, 44.3 ± 12.7 % humidity), and MIDS (22.9 ± 1.2ºC, 41.3 ± 15.7 % humidity) trials (*p* values > 0.300 for all comparisons). Moreover, the order effects of time trial performance when comparing V2 to V3 (p = 0.954) or V2 to V4 (p = 0.272) were not significant.

### 3.3. Metabolic cart variables during the 5-kilometer time trial between conditions

**Figure 4.**
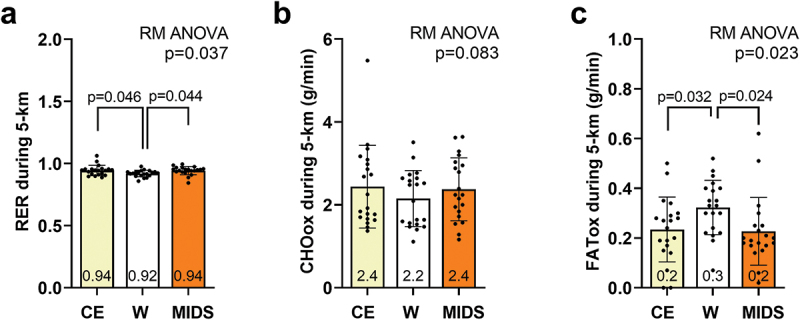
Gas exchange variables during 5-km time trials. repeated measures (RM) ANOVAs indicated that significant differences between trials existed for respiratory exchange ratio (RER, panel a) values and fat oxidation (FATox, panel c) values during the 5-km time trials. No significant difference was found for carbohydrate oxidation (CHOox, panel b). All data are presented as mean ± standard deviation values, individual respondent data are presented as data points within each bar, and the mean scores are presented at the bottom of each bar.

Gas exchange variables during 5-km time trials are presented in Figure 4. A significant difference between trials existed for 5-km respiratory exchange ratio (RER) (*p* = 0.037; Figure 4a), and post hoc tests indicated values were lower during the W versus other trials. Although a significant difference was not observed for estimated carbohydrate oxidation rates between trials (*p* = 0.083; Figure 4b), a significant difference between trials existed for estimated fat oxidation rates (*p* = 0.037; Figure 4c), and post hoc tests indicated values were lower during the W versus other trials.

### 3.4. Blood measures

**Figure 5.**
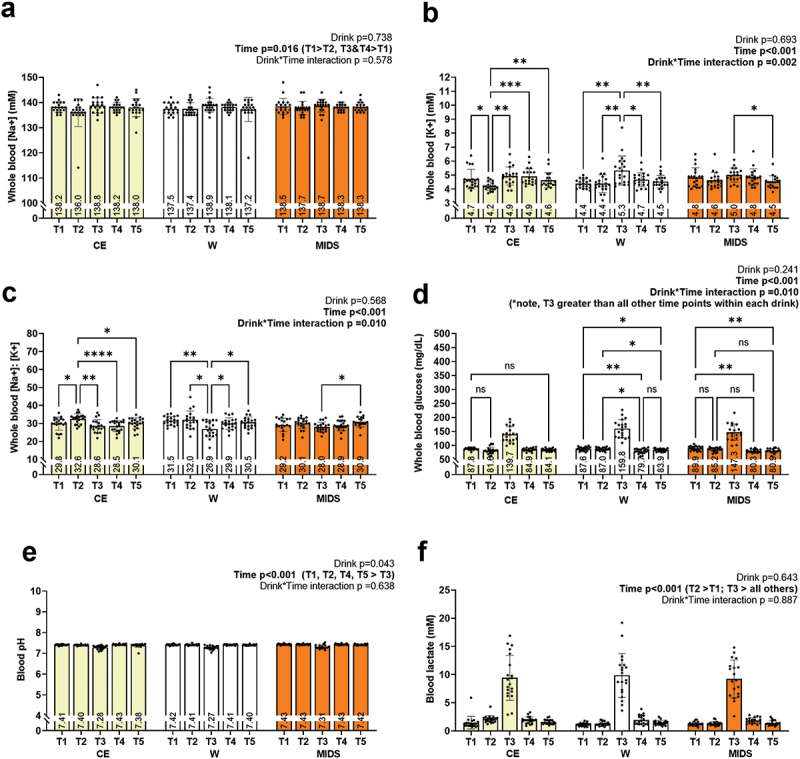
Blood electrolytes, blood glucose, and other markers. no significant drink*time interactions existed for blood sodium (panel a), blood pH (panel e), or blood lactate (panel f). Significant drink*time interactions did exist for blood potassium (panel b) concentrations as well as sodium: potassium levels (panel c). Post hocs indicated that these levels were best maintained in the MIDS condition versus CE and W conditions. A significant drink*time interaction also existed for blood glucose concentrations (panel d), and post hocs indicated that post-exercise levels were better maintained in the CE condition versus the MIDS and W conditions. Significance symbols: *, *p* < 0.05; **, *p* < 0.01; ***, *p* < 0.005; ****, *p* < 0.0001.

Blood measures are presented in Figure 5. For the sake of simplicity, within-trial time points for this section are presented as follows in-text: T1, prior to drink consumption; T2, 45 min following drink consumption; T3, immediately post-exercise; T4, 60 mins post-exercise; T5, 120 mins post-exercise. Blood Na^+^ (Figure 5a), pH (Figure 5e) and lactate (Figure 5f) displayed no significant drink*time interactions; however, Na^+^, pH, and lactate displayed main effects of time (Na^+^: T1 > T2, T3&T4 > T1, *p* = 0.016; pH: T1, T2, T4, T5 > T3, *p* < 0.001; lactate: T2 > T1, T3 > all others, *p* < 0.001).

Blood K^+^ displayed a significant drink*time interaction (*p* = 0.002; Figure 5b) and a significant main effect of time. During the CE condition, there was a significant decrease in K^+^ from T1 to T2, a significant increase in K^+^ from T2 to T3, and values remained relatively constant for the remainder of the trial. During the W condition, K^+^ remained constant for T1 and T2 and displayed a significant increase between T2 and T3 then significantly decreased between T3 and T4 and remained constant for T4 and T5. During the MIDS condition, the only effect of time was displayed between T3 and T5 where there was a significant decrease in K^+^ between T3 and T5.

Sodium/potassium ratio (Na^+^/K^+^) displayed significant drink*time interaction (*p* = 0.010; Figure 5c), and a significant main effect of time (*p* < 0.001). During the CE condition, Na^+^/K^+^ increased between T1 and T2, decreased between T2 and T3, and remained suppressed for T4, and T5. During the W condition, Na^+^/K^+^ remained unaltered during T1 and T2, decreased from T2 and T3, then increased at T4 and remained elevated at T5. During the MIDS condition, the only difference observed for Na^+^/K^+^ was T3 values being lower than T5 values.

Glucose displayed significant drink*time interaction (*p* = 0.010; Figure 5c), and a significant main effect of time (*p* < 0.001). During the CE condition, glucose decreased from T1 to T2, increased from T2 to T3, then decreased at T4 and remained decreased at T5. During the W condition, glucose remained unaltered during T1 and T2, increased from T2 to T3, decreased from T3 to T4, and increased from T4 to T5. During the MIDS condition, glucose decreased from T1 to T2, increased from T2 to T3, decreased from T3 to T4, then remained unaltered between T4 and T5.

### 3.5. Hydration markers

**Figure 6.**
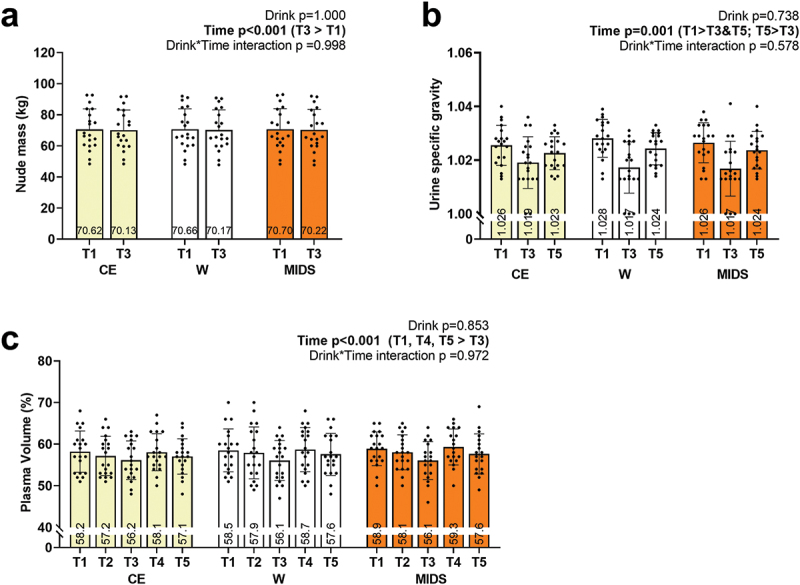
Hydration markers. as seen above, no significant drink*time interactions existed for nude mass (panel a), urine specific gravity (panel b), or plasma volume changes (panel c). However, significant main effects of time existed for all markers whereby USG (for instance) was improved following exercise (which was likely due to the consumption of the drink prior to exercise). Nude mass also decreased post-exercise, regardless of drink), and this was likely due to sweat loss.

Hydration markers are presented in Figure 6. Nude mass was recorded during T1 and T3. There was no drink*time interaction, but the main effects of time were present. T1 was significantly greater than T3 (*p* < 0.001; Figure 6a). USG was recorded during T1, T2, and T5. T1 was significantly greater than T3 (*p* < 0.001) and T5 (*p* = 0.001), and T5 was significantly greater than T3 (*p* = 0.001). Plasma volume was recorded during all timepoints. T1, T2, T4, and T5 were significantly greater than T3 (p-value ranges: 0.011 to < 0.001).

### 3.6. Cramp prevalence

**Figure 7.**
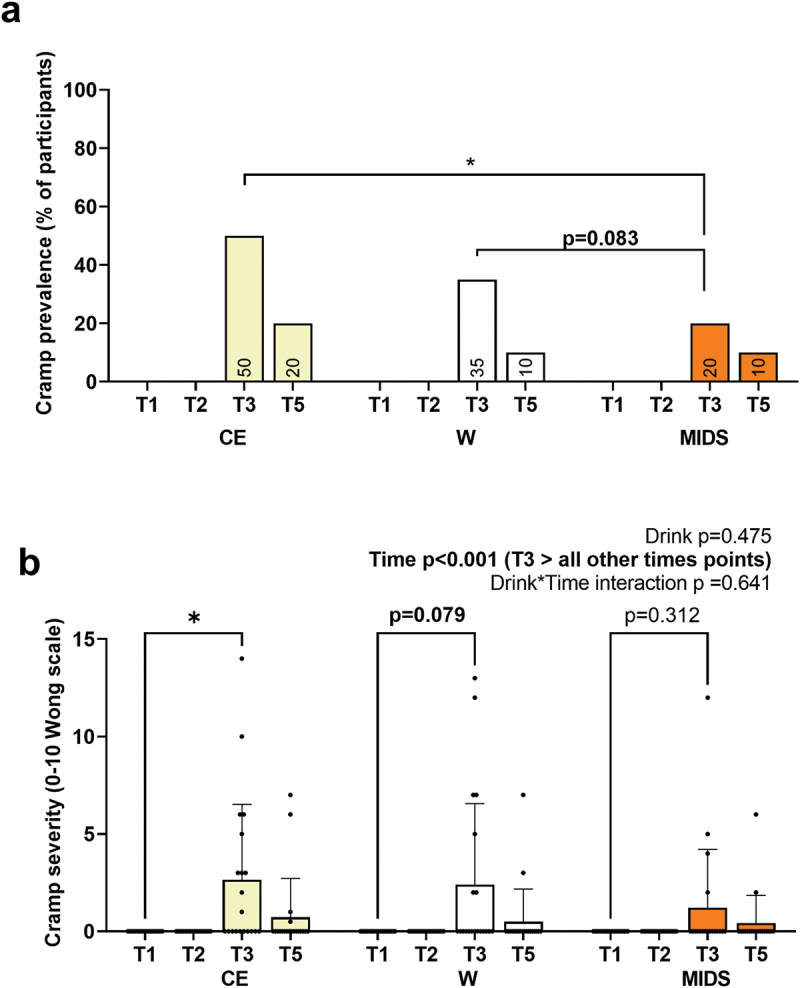
Cramping data. the least number of participants (on a percentile basis) reported post-exercise cramping during the MIDS condition (panel a). When examining the severity of cramping (using the Wong-Baker scale of 0–10), post-exercise values were significant in the CE condition and trended for the W condition, but not the MIDS condition (panel b). Significance symbol: *, *p* < 0.05.

Cramp prevalence and severity measures (based on a 0–10 Wong scale) are presented in Figure 7. For cramp prevalence (Figure 7a), repeated measures ANOVAs were performed using post-exercise (T3) and 120-min post-exercise (T5) data since no cramps were reported prior to the time trials. Notably, more participants reported cramping immediately post-exercise during the CE versus MIDS condition (50% versus 20%, respectively; dependent samples t-test *p* = 0.030, Wilcoxon *p* = 0.041). More participant cramping immediately post-exercise also approached statistical significance (according to parametric statistical testing) during the W versus MIDS condition (35% versus 20%, respectively; dependent samples t-test *p* = 0.083, Wilcoxon *p* = 0.149).

There was a significant main effect of time for cramp severity (T3 > all other time points, *p* < 0.001; Figure 7b). Further, although no significant interaction was noted, Tukey’s post hoc tests indicated that cramp severity at T3 versus T1 was elevated during the CE condition (Tukey *p* = 0.029, Wilcoxon *p* = 0.006) and trended upward in the W condition (Tukey *p* = 0.079, Wilcoxon *p* = 0.022), but not the MIDS condition (Tukey *p* = 0.312, Wilcoxon *p* = 0.100).

### 3.7. Abbreviated POMS scores

**Figure 8.**
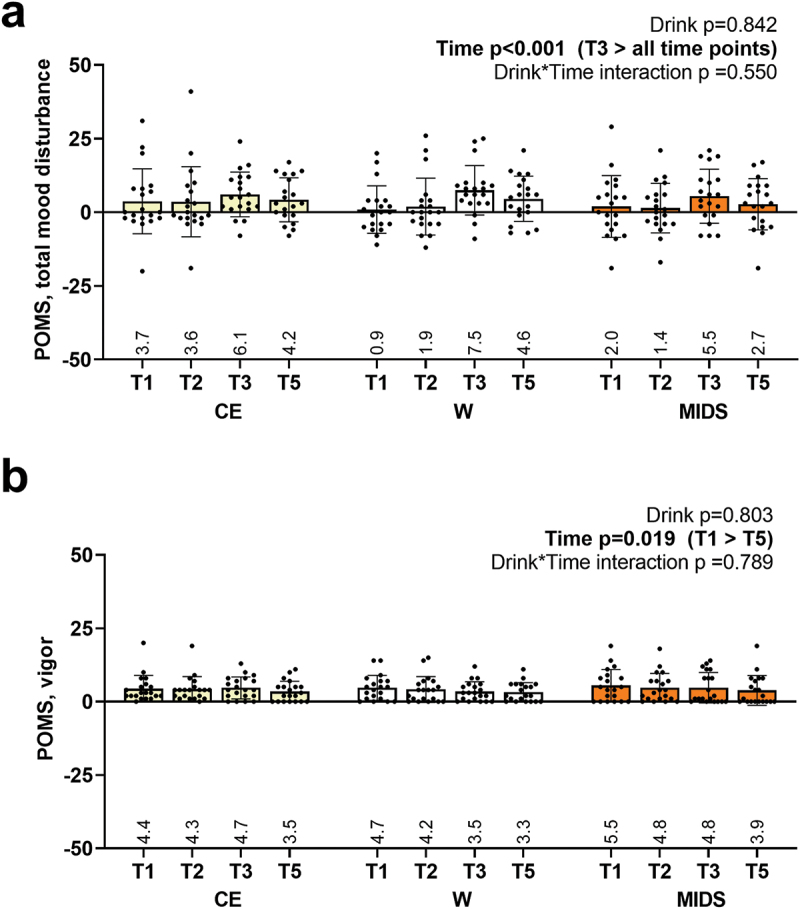
POMS scores. as seen above, no significant drink*time interactions existed for POMS-total mood disturbance (panel a) or POMS-vigor (panel b). However, significant main effects of time existed for these subjective measures.

Abbreviated POMS scores are presented in Figure 8. There were no drink*time interactions observed, but main effects of time were present. For total mood disturbance, T3 was significantly greater than all other time points (p-value ranges: 0.005 to < 0.001; Figure 8a). For vigor, only T1 was greater than T5 (*p* = 0.019; Figure 8b).

### 3.8. Hemodynamics

**Figure 9.**
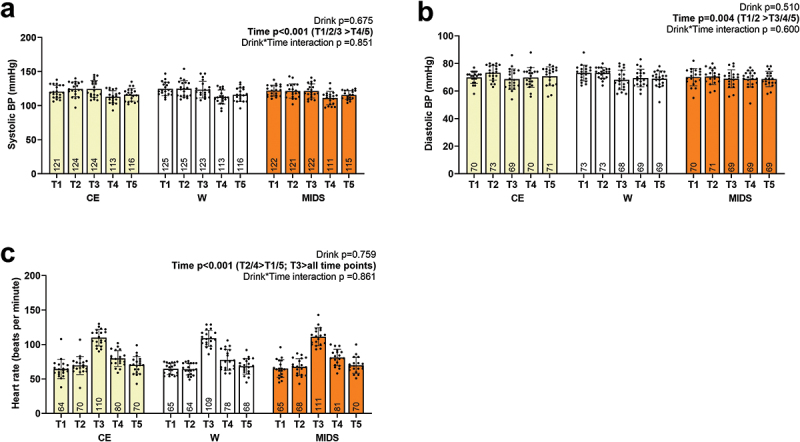
Hemodynamics. as seen above, no significant drink*time interactions existed for systolic blood pressure (panel a), diastolic blood pressure (panel b), or heart rate (panel c). However, significant main effects of time existed for all markers.

Hemodynamics are presented in Figure 9. There were no drink*time interactions observed, but main effects of time were present. For systolic BP (Figure 9a), T1, T2, and T3 were greater than T4 and T5 (*p* < 0.001). For diastolic BP (Figure 9b), T1 and T2 were greater than T3, T4, and T5 (*p* = 0.003). For HR (Figure 9c), in beats per min, T2 and T4 were significantly greater than T1 and T5, and T3 was significantly greater than all other time points (*p* = 0.003).

## 4. Discussion

We aimed to examine the effects of a MIDS (XTEND® Healthy Hydration; Nutrabolt, Austin, TX, USA) on 5-km time trial performance, hydration markers, cramp prevalence/severity, and perceptions of mood states compared to a conventional carbohydrate-electrolyte beverage (GATORADE® Thirst Quencher; PepsiCo, Purchase, NY, USA) and distilled water. The main findings from this study are: i) that the MIDS had no effect on 5-km time trial performance, ii) K^+^ and Na^+^/K^+^ displayed the least amount of change between timepoints during the MIDS condition, and iii) whole blood glucose being elevated the greatest during the water condition. Moreover, interesting effects existed between conditions for cramping and warrant further discussion.

Although prior studies have demonstrated the potential benefits of some of these ingredients to enhance exercise performance [19–23], the other MIDS ingredients such as the specific electrolyte blend have limited ergogenic efficacy data. Snell et al. [24] reported ingesting an electrolyte-containing beverage improves exercise performance following exercise-induced dehydration bouts compared to Gatorade and a calorie/electrolyte-free beverage. Moreover, certain studies suggest that the ingestion of deep ocean water, which contains a modest electrolyte profile, enhances aerobic recovery from dehydrated states compared to commercial drinking water [25]. The current study displayed no differences in time trial performance between conditions. Our null findings differ from those of Snell and colleagues, but aligns with studies by Lutsch et al. [26] and Desroches et al. [27] who examined the effects of different dietary supplements on 5-km time trial performances compared to a placebo. The investigation by Lutsch and colleagues utilized a different pre-workout supplement containing 150 milligrams of caffeine, 1.6 grams of beta-alanine, and 1 gram of arginine, rather than the MIDS administered herein. These authors reported that 5-km time trial performance was not affected in 10 aerobically trained men relative to a placebo condition. The investigation by Desroches and colleagues administered cold water and glycerol to 10 recreationally active participants to induce hyperhydration. Although hyperhydration was achieved, this strategy did not improve 5-km time trial performance relative to the placebo condition. The lack of performance benefits between conditions for the two aforementioned studies (as well as our study) could be attributed to the absence of caffeine in any of the conditions, as supplements that have been shown to improve 5-km time trial performance contained caffeine [28,29]. Moreover, there is little evidence that consuming carbohydrates/electrolytes during activities less than 45 min provides significant performance benefits over water alone [30,31]. Hence, given that the 5-km time trials lasted ~28 min on average, these are plausible reasons as to why CE and MIDS did not improve time trial performance compared to W. Moreover, our findings may not align with Snell et al. [24] given that the current participants were not pre-exhausted prior to performing the 5-km time trials. Hence, future research is needed to determine if the current MIDS can mitigate exercise performance after preexisting bouts.

Considering the utility of assessing plasma Na^+^ as a surrogate marker of hydration status and its implications on performance [32,33], an interesting finding was the ability of MIDS to better maintain whole blood K^+^ and Na^+^/K^+^. MIDS contains various electrolytes (Na^+^, K^+^, Ca^2+^, Mg^2+^, Cl^−^, and PO4^3-^) which are critical for maintaining total body water and hydration [34–38]. Whole body K^+^ showed fluctuations between timepoints during the CE and W and conditions but was only different between two timepoints (T3 and T5) during the MIDS condition. These data indicate that the MIDS amino acid and electrolyte blend could be superior to CE in maintaining blood electrolyte levels. Although reasons for this are unknown, it is notable that on a per 16 fl. oz. basis, the MIDS contained greater amount of Na^+^ and K^+^ (Na^+^: 450 mg, and K^+^: 380 mg) than CE (Na^+^: ~200 mg, and K^+^: ~63 mg). Hence, the increased electrolyte load during the MIDS condition may have produced these blood profiles. Blood Na^+^/K^+^ also showed the least amount of change between conditions (only T3 and T5) during the MIDS condition, which again supports this contention. Na^+^/K^+^ is a measure that is mainly associated with the maintenance of normotensive blood pressures [39]. Further, the blood Na^+^/K^+^ ratio may be indicative of less endothelial stress during intense exercise [40]. However, it should be noted that there were no interactions between conditions for the hemodynamic variables, and markers of endothelial stress (e.g. circulating endothelial microparticles) or endothelial function were not obtained herein. Thus, more research is needed to determine how these physiological responses are altered with longer endurance exercise trials.

Another notable finding was that MIDS was able to reduce cramp incidence and severity relative to the other two conditions. The cramp data was secondary to the performance and blood outcomes, and it is unexpected that MIDS was superior in reducing cramp outcomes given that the 5-km trials were brief and occurred in an ambient environment. Thus, this was an unanticipated finding. This may have been due to the greater maintenance of blood electrolytes, as it has been suggested that blood K^+^ and Na^+^ imbalances during strenuous exercise may contribute to exercise-induced cramping [41]. However, other literature indicates that factors such as previous cramp history, the degree of muscle activation during exercise, or exercise intensity are more influential in predicting cramp prevalence during exercise [42,43]. Moreover, a recent and comprehensive review on exercise-induced muscle cramps indicates two prevalent mechanistic hypotheses exist including the “dehydration or electrolyte depletion mechanism” and the “neuromuscular mechanism” [44]. The authors of this review also posited that, based on the available evidence to date, the “neuromuscular mechanism” is the more likely candidate for exercise-induced muscle cramps and that this mechanism results from prolonged exercise leading to motoneurons receiving afferent signals that result in hyperexcitability. Testing as to whether MIDS affected neural mechanisms related to cramping was beyond the current scope of this study and examining muscle afferent activity in humans (while possible) is cumbersome and technically challenging [45]. Notwithstanding, the positive effects that MIDS had on reducing muscle cramps are intriguing and warrant future research wherein longer endurance event protocols in hotter environments are employed.

What is finally notable is that substrate utilization was similar between the CE and MIDS conditions despite the latter lacking an appreciable amount of carbohydrates. Although this seems paradoxical, there are plausible explanations. First, several electrolytes act to stimulate enzymes involved in glycogen breakdown, and the MIDS electrolyte profile was more robust relative to the CE beverage. For instance, Ca^2+^ increases phosphorylase activity which mobilizes glycogen [46]. Since the participants were likely not glycogen deprived during any of the conditions, and muscle glycogen is readily utilized during aerobic activities, this mechanism could have been operative. Mg^2+^ is a cofactor hexokinase and phosphofructokinase [47]. K^+^ facilitates glucose uptake in muscle cells via GLUT4 transporters and is a co-factor for pyruvate dehydrogenase [48]. Additionally, betaine has been shown to augment Akt signaling in skeletal muscle [49], and while speculative, this too could have transient effects in increasing glycolytic flux during exercise. Finally, the 2.3 g of amino acids per MIDS may have affected carbohydrate oxidation. However, we posit this is less likely given that the only glucogenic amino acid in MIDS is L-alanine, blood glucose levels were not atypically affected during the MIDS compared to the other conditions (Figure 5d), and others have shown that a 15 g dose of amino acids (i.e. ~6.5× the MIDS dose) does not affect blood glucose concentrations. Regardless of mechanistic plausibility, MIDS seemingly favors carbohydrate oxidation during exercise. Given that carbohydrate oxidation has been deemed as a prominent determinant for athletic success during aerobic events [50], the consumption of MIDS (rather than glucose-containing drinks) could be a viable strategy in those desiring to optimize carbohydrate oxidation rates during aerobic exercise bouts lasting ~ 30–60 min.

Certain limitations of the study should be noted. First, while participants met our aerobic capacity criteria, they were not elite runners. Hence, these data should be interpreted in that regard. Additionally, while there was no statistical indication of an order effect on 5-km performance, there is the potential that some participants may have improved performance from visits 2–4 which could potentially confound the time trial data. Finally, and as mentioned, our data do not extrapolate to hotter conditions whereby rehydration for mitigation of performance decrements and cramping is more relevant. Hence, it remains to be determined how the current MIDS supplement affects performance or cramp outcomes therein.

## 5. Conclusions

Although MIDS did not affect 5-km time trial performance in recreationally trained individuals, it did better maintain post-exercise blood K^+^ and the Na^+^/K^+^ ratio compared to CE and W. Additionally, post-exercise cramp prevalence and severity were the lowest in the MIDS versus other conditions. Further research is warranted to confirm if MIDS can extend exercise performance during longer endurance exercise bouts and/or contribute to greater hydration perseverance under physiologically stressful conditions such as hot environments.

## Data Availability

Raw data related to the current study outcomes will be provided upon reasonable request by emailing the corresponding author (mdr0024@auburn.edu).
